# Supplementary material for: An 8q24 Gain in Pancreatic Juice Is a Candidate Biomarker for the Detection of Pancreatic Cancer
Source: Int J Mol Sci. 2023 Mar 7;24(6):5097. doi: 10.3390/ijms24065097 (PMC10049288; doi:10.3390/ijms24065097)
Supplement: Supplementary file 1 [file ijms-24-05097-s001.zip › ijms-2245982-supplementary.pdf]

Supplemental Table S1: in- and exclusion criteria per prospective cohort study.

Prospective cohort	Inclusion criteria	Exclusion criteria
**KRASPanc**	Patients who undergo an EUS for (suspected) PDAC either as part of a diagnostic process or fiducial placement prior to radiotherapeutic treatment.	Age <18 years
**CAPS**	Individuals who, after evaluation by a clinical geneticist, have an estimated 10-fold increased risk of developing PDAC, this includes: (1) Carriers of a gene mutation in *CDKN2A* or *STK11*, regardless of the family history of pancreatic cancer (2) Carriers of a gene mutation in *BRCA1*, *BRCA2*, *p53*, or Mismatch Repair Gene with a family history of PDAC in ≥ 2 family members. (3) Familial PDAC (FPC) kindreds, defined as individuals with at least (1) 2 first-degree relatives (FDR) with PDAC, (2) 3 relatives with pancreatic cancer, either FDR or second degree relative (SDR), or (3) 2 SDR relatives with pancreatic cancer of which at ≥1 was <50 years at time of diagnosis.	Age <18 years, personal history of pancreatic cancer, individuals unable to provide informed consent, severe medical illness, PRSS1 gene carrier, contra-indication for EUS due to anatomic abnormalities/surgery
**PACYFIC**	Individuals with a suspected neoplastic pancreatic cyst (either newly or previously diagnosed, or previously operated upon) for which cyst surveillance is warranted, according to the treating physician.	Age <18 years, history of chronic pancreatitis, suspected pseudocyst (simple, thin-walled cyst that developed in the course of acute pancreatitis, as documented by sequential imaging studies), suspected serous cystadenoma (typical microcystic lesion with lobulated outlines, a calcified central scar and/or cyst fluid CEA levels < 5ng/ml), Von Hippel-Lindau disease, and limited life expectancy (<2 years).

Supplemental Figure S1: H&E staining of tumor slides showing the cancer cellularity at time of resection for P1 (A; PC), P3 (B; PC) and P4 (C-D; HGD). No slide was available of P2 at time of writing. PC = pancreatic cancer; HGD = high-grade dysplasia.

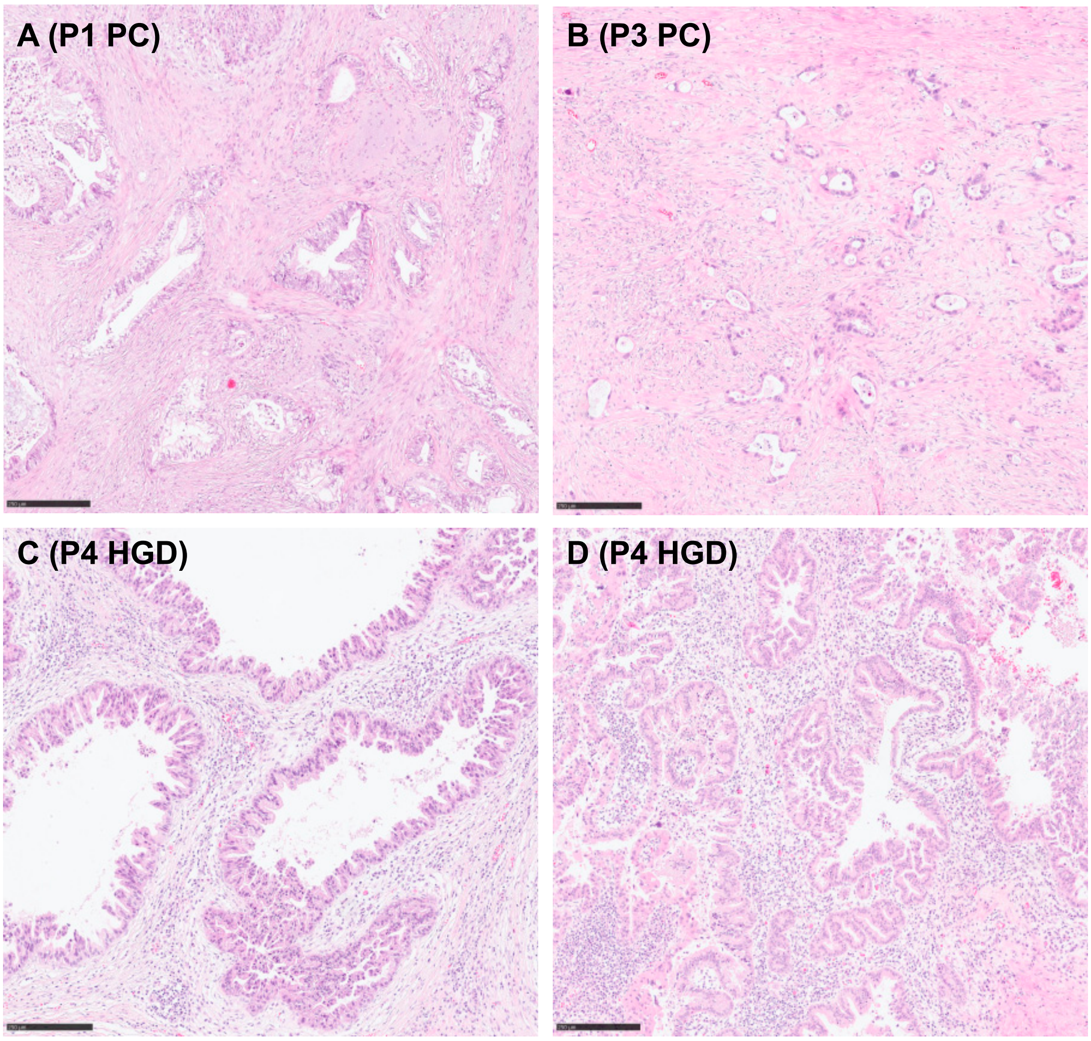

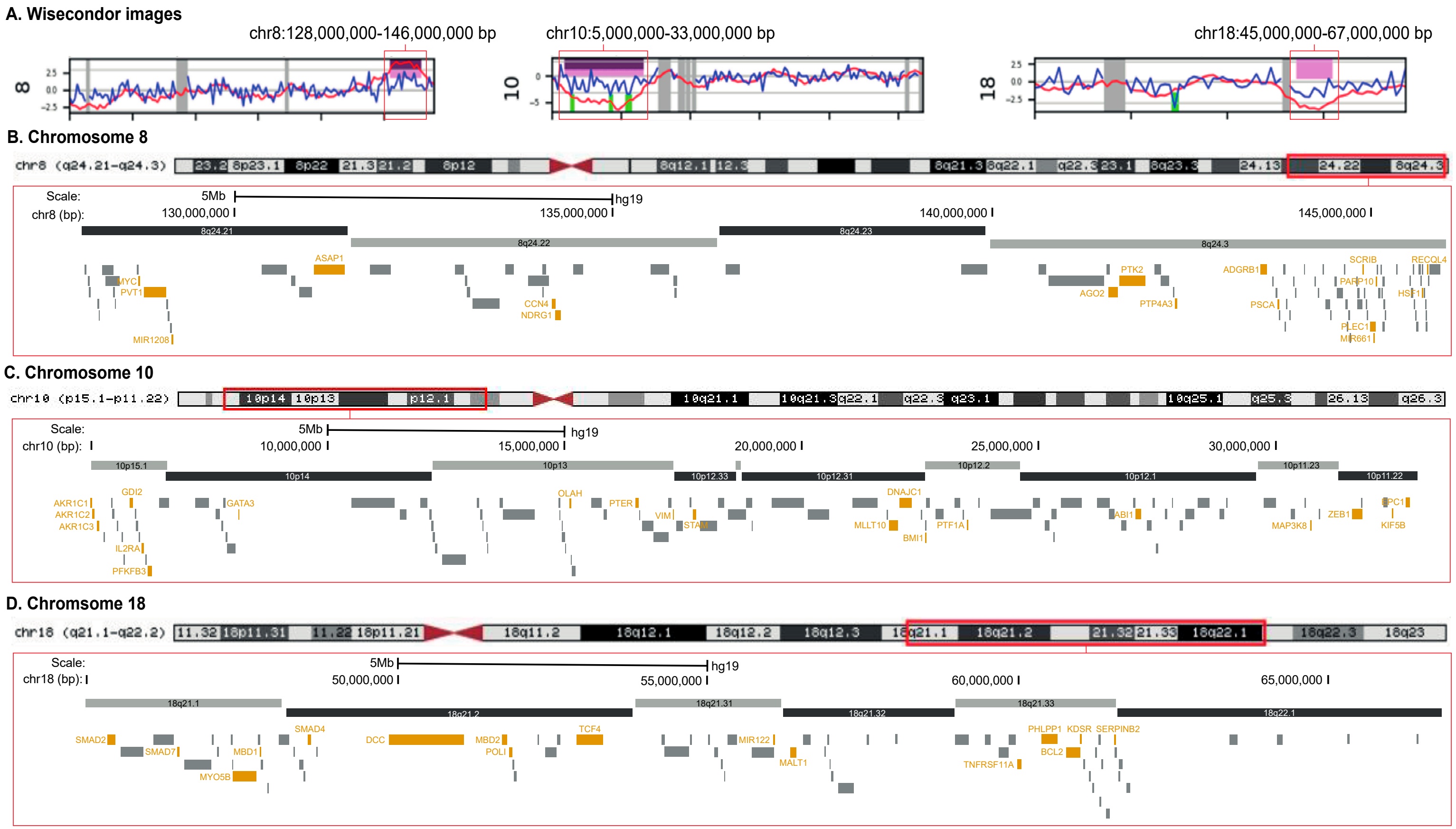

Supplemental Figure S2: An overview of copy number variations seen in the 8q subgroup and genes located on the aberration segments. A. Snapshot of the Wisecondor images showing an 8q gain and 10p loss and 18q loss. Aberrant segments are marked with a red box. Purple = significant copy number variations called by the software. Pink = copy number variations of uncertain significance. **B-C** Genes located on the aberrant chromosome segments. Yellow = genes associated with cancer development according to previous literature.

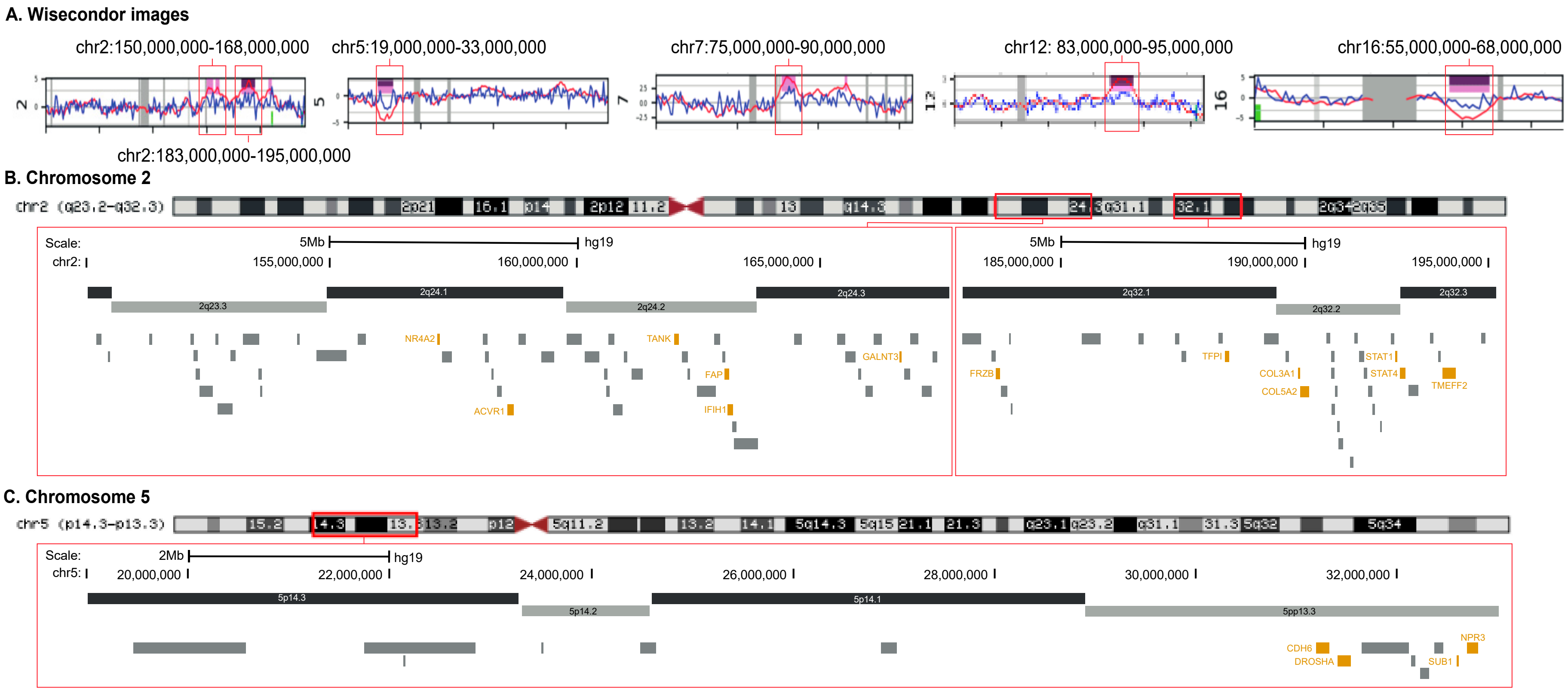

Supplemental Figure S3: An overview of copy number variations seen in the 2q-5p subgroup and genes located on the aberration segments. A. Snapshot of the Wisecondor images showing a 2q gain and 5p loss (with or without 7q gain, 12q gain or 16q loss). Aberrant segments are marked with a red box. Purple = significant copy number variations called by the software. Pink = copy number variations of uncertain significance. **B-F** Genes located on the aberrant chromosome segments. Yellow = genes associated with cancer development according to previous literature.

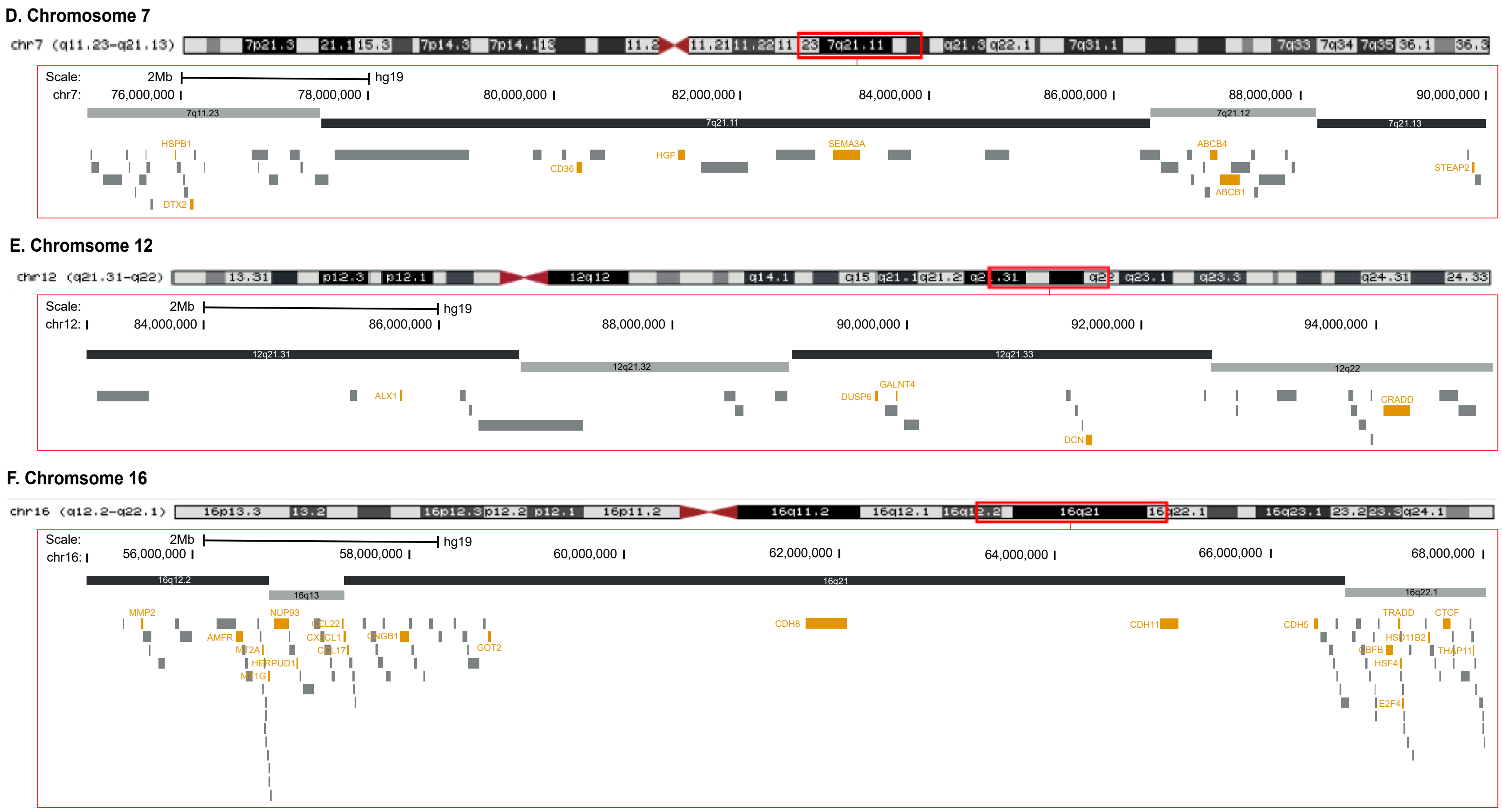

Supplemental Figure S3: An overview of copy number variations seen in the 2q-5p subgroup and genes located on the aberration segments. A. Snapshot of the Wisecondor images showing a 2q gain and 5p loss (with or without 7q gain, 12q gain or 16q loss). Aberrant segments are marked with a red box. Purple = significant copy number variations called by the software. Pink = copy number variations of uncertain significance. **B-F** Genes located on the aberrant chromosome segments. Yellow = genes associated with cancer development according to previous literature.
